# Supplementary material for: Correspondence between neuroevolution and gradient descent
Source: Nat Commun. 2021 Nov 2;12:6317. doi: 10.1038/s41467-021-26568-2 (PMC8563972; doi:10.1038/s41467-021-26568-2)
Supplement: Supplementary file 1 — Supplementary Information [file 41467_2021_26568_MOESM1_ESM.pdf]

Supplementary Figures for
"Correspondence between neuroevolution and gradient descent"

Stephen Whitelam, Viktor Selin, Sang-Won Park, and Isaac Tamblyn

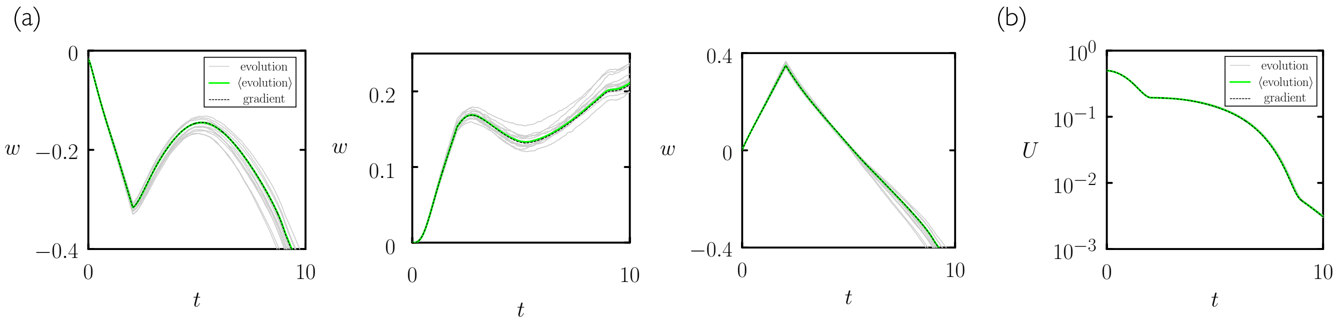

Supplementary Fig. 1. As Fig. 1(a) and (c), but with simulations initiated from a different randomly-chosen neural network; the neuroevolution-gradient descent correspondence is again apparent. Here we show three weights (a) and the loss (b).

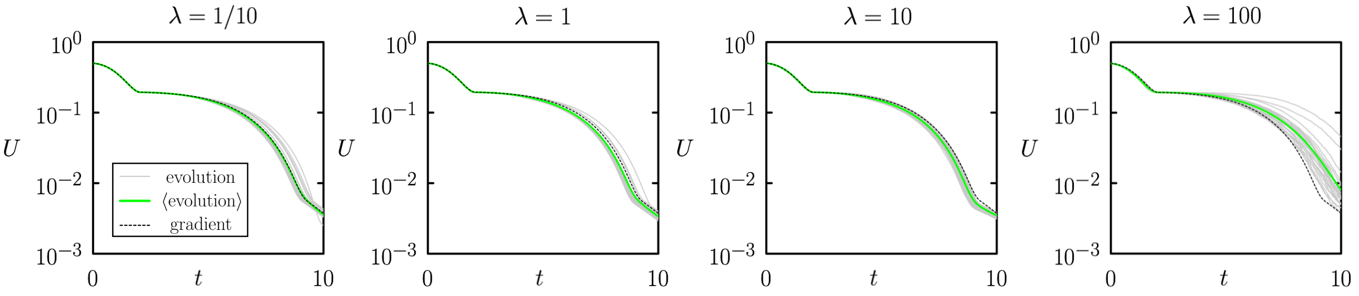

Supplementary Fig. 2. As Fig. 2(a), but showing the evolution of the loss function $U(\mathbf{x})$ with time. For small mutations λ [see Eq. (14)] the neuroevolution-gradient descent correspondence is apparent; for larger mutations the dynamics of neuroevolution and gradient descent are different, but neurevolution can still learn. For sufficiently large mutations, neuroevolution will cease to learn.

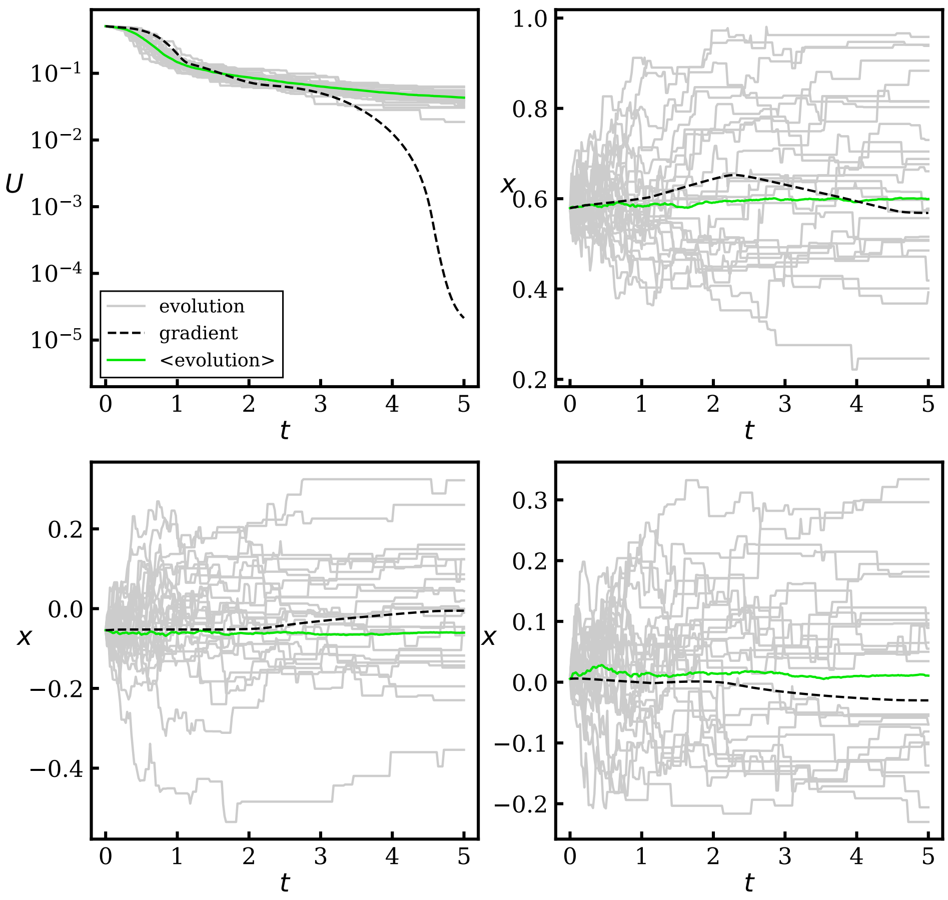

Supplementary Fig. 3. As Fig. 3, but for mutations large enough ($\lambda = 100$) that correspondence has broken down.

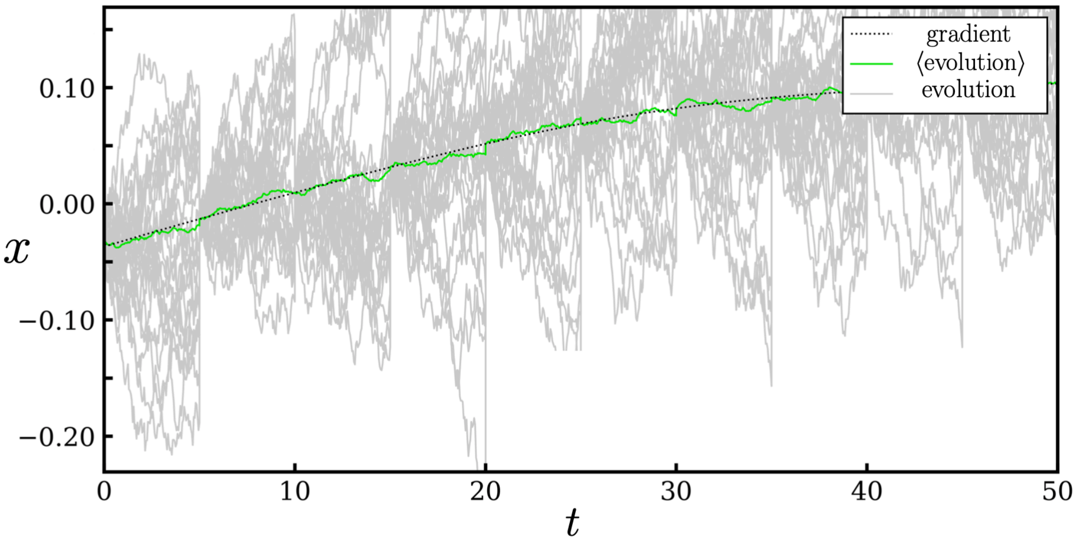

Supplementary Fig. 4. Supplement to Fig. 6: Illustration of the neuroevolution-gradient descent correspondence for the case of finite β, with periodic resetting (every 5 time units) of the neurevolution trajectories. The panel shows one weight of the network.
